# The Impact of Comorbid Chronic Pain on Pharmacotherapy for Veterans with Post-Traumatic Stress Disorder

**DOI:** 10.3390/jcm12144763

**Published:** 2023-07-19

**Authors:** Alessandra A. Pratt, Katherine Hadlandsmyth, Michelle A. Mengeling, Emily B. K. Thomas, Kelly Miell, Sonya B. Norman, Brian C. Lund

**Affiliations:** 1 Center for Access & Delivery Research and Evaluation (CADRE), Iowa City VA Health Care System, 601 Highway 6 West, Building 42, Iowa City, IA 52246, USA; 2 VA Office of Rural Health (ORH), Veterans Rural Health Resource Center-Iowa City (VRHRC-IC), Iowa City VA Health Care System, 601 Highway 6 West, Iowa City, IA 52246, USA; 3 Department of Anesthesia, University of Iowa Carver College of Medicine, 200 Hawkins Drive, Iowa City, IA 52242, USA; 4 Department of Internal Medicine, University of Iowa Carver College of Medicine, 200 Hawkins Drive, Iowa City, IA 52242, USA; 5 Department of Psychological and Brain Sciences, University of Iowa College of Liberal Arts and Sciences, 340 Iowa Ave, Iowa City, IA 52246, USA; 6 National Center for PTSD, 215 North Main Street, White River Junction, VT 05009, USA; 7 Department of Psychiatry, University of California San Diego School of Medicine, 9500 Gilman Drive, La Jolla, CA 92093, USA; 8 Department of Biostatistics, University of Iowa College of Public Health, 145 N Riverside Drive, Iowa City, IA 52242, USA

**Keywords:** PTSD, chronic pain, pharmacoepidemiology, Veterans

## Abstract

Objective: Chronic pain can worsen PTSD symptomatology and may increase the risk of the prescription of multiple central nervous system (CNS)-active medications. The objective is to determine the impact of chronic pain on the number of CNS medications, including psychiatric medications, as well as the amount of medication changes. Methods: Veterans Affairs (VA) administrative data were used to identify VA-served Veterans with PTSD (N = 637,428) who had chronic pain (50.3%) and did not have chronic pain (49.7%) in 2020. The outcomes included the number of changes in psychiatric medications and the number of currently prescribed CNS-active mediations during a one-year observation period. Results: The number of changes in psychiatric medications was significantly higher for those with chronic pain (mean (M) = 1.8) versus those without chronic pain (M = 1.6) (Z = 38.4, *p* < 0.001). The mean number of concurrent CNS-active medications were significantly higher for those with chronic pain (M = 2.7) versus those without chronic pain (M = 2.0) (Z = 179.7, *p* < 0.001). These differences persisted after adjustment for confounding factors using negative binomial regression. Conclusions: Veterans with comorbid chronic pain and PTSD are at increased risk for a higher number of medication changes and for receiving CNS-active polytherapy.

## 1. Introduction

Comorbid post-traumatic stress disorder (PTSD) and chronic pain are common among Veterans [1,2,3]. Chronic pain can amplify the symptoms of PTSD and complicate treatment, resulting in a greater use of healthcare services, which may be caused by poorer response to pain treatment [4,5,6,7]. Veterans with comorbid chronic pain and PTSD report having a poorer quality of life and have worse pain and psychological outcomes than those without concurrent disorders [8,9,10]. Specifically, Veterans with both PTSD and chronic pain have increased pain intensity or severity, can have pain-related disability, pain catastrophizing, and an increase in depressive and anxiety symptom severity [11,12,13,14,15,16]. Chronic pain can also result in greater psychiatric symptom instability among Veterans with PTSD [9,10]. As such, comorbid chronic pain and PTSD may result in an accumulation of multiple medications to treat these conditions [17]. Specifically, severe PTSD symptoms, magnified by chronic pain, may require more frequent psychotropic medication changes across time. However, it has not been clearly demonstrated whether those with comorbid chronic pain and PTSD receive more CNS medications or have more frequent psychotropic medication changes than those with PTSD but without chronic pain.

The siloed management for PTSD and chronic pain can result in the prescription of multiple concurrent central nervous system (CNS)-active medications [17,18]. Polytherapy with CNS-active medications can increase the risk of overdose mortality, suicide-related behaviors, and unintentional death [19]. Other effects of polytherapy can include the following: increased healthcare costs, adverse drug events, drug interactions, medication non-adherence, individual functional decline, cognitive impairment, falls, urinary incontinence, and change in nutritional status [20,21,22]. Polytherapy among Veterans with comorbid PTSD and chronic pain may result from an additive effect of multiple conditions, or it may be impacted by increased difficulties in managing psychiatric symptoms in the presence of comorbid chronic pain.

The current study aims to examine the impact of comorbid chronic pain on Veterans with PTSD via psychopharmacological prescription patterns. Specifically, this study aims to (1) determine the impact of chronic pain on a number of psychiatric medication changes across time (as an indicator of treatment instability) among Veterans with PTSD, and (2) determine the impact of comorbid chronic pain on the risk of multiple concurrent CNS-active medication prescriptions among Veterans with PTSD. We hypothesized that Veterans with chronic pain would have more psychiatric medication changes across time and have a higher number of concurrent CNS-active medications.

## 2. Methods

### 2.1. Data Sources

National Veterans Affairs (VA) administrative data from the VA Corporate Data Warehouse were used for this study. The presence of mental health and other medical comorbidities were determined using international classification of disease (ICD) codes from inpatient and outpatient encounters. Drug exposure was assessed using outpatient pharmacy dispensing data. The current study is an operations-supported quality improvement project determined not to constitute human subjects research by the local Institutional Review Board.

### 2.2. Patients

Veterans with PTSD were identified using ICD-9 and ICD-10 codes (309.81 and F43.1X) from inpatient and outpatient encounters. Patients were required to have at least one inpatient hospitalization coded for PTSD during 2020, or at least one PTSD-coded outpatient encounter during 2020 and a second PTSD-coded encounter within the past 730 days [23,24].

### 2.3. Outcomes

Exposure to two groups of medications was assessed. First, medications with CNS activity were identified using VA drug classification, as defined by Collett and colleagues [17,19]. Second, a subset of these medications typically used for psychiatric indications was examined, and was limited to antidepressants, antipsychotics, anticonvulsants, benzodiazepines, z-drug hypnotics, stimulants, and lithium (Supplemental Table S1). Three specific medications from these classes, gabapentin, topiramate, and duloxetine, were not considered in the psychiatric medication subset, as these agents are commonly used for the management of pain.

Drug exposure was assessed during the calendar year 2021, the year following patient selection, to ensure that diagnoses of PTSD and chronic pain preceded the outcome. The outcome measure for Aim 1 was the number of changes in psychiatric medication during the observation year. This was assessed by determining which psychiatric medications were active on the first day of the observation year, which were active on the last day of the observation year, and other psychiatric medications dispensed throughout the observation year. Medications present in the baseline regimen, but not in the follow-up regimen, were considered discontinued and counted as one change. Conversely, medications present at follow-up but not at baseline were considered new medications and counted as one change. Medications dispensed during the year, but not present in either regimen, were considered to have been started and then stopped and counted as two changes. The outcome measure for Aim 2 was the maximum number of CNS-active medications received concurrently at any point during the observation year, using previously established methodology [17]. Both outcome measures relied on longitudinal prescription histories, where medications were considered active on any given day during this period based on cabinet supply methodology [17,25]. Briefly, this approach estimates the day’s supply on hand for each calendar day during a specified time interval, with adjustments for carrying forward oversupply for early refills and allowable nonadherence.

### 2.4. Analysis

The focus of the analysis was to determine whether the presence or absence of chronic pain was associated with two clinically relevant measures of drug exposure including (1) the number of psychiatric medication changes and (2) the maximum number of concurrent CNS-active medications over a one-year observation period. As both outcome measures were discrete counts with low frequencies and not expected to be normally distributed, bivariate associations with chronic pain were examined using the nonparametric Wilcoxon rank sum test. Chronic pain was identified using Tian’s criteria [26], modified to include ICD-10 codes [27]. To meet criteria for chronic pain, patients were required to meet one of the following 3 criteria: 2 outpatient encounters separated by ≥30 days with a diagnosis code likely indicating chronic pain; at least 1 encounter coded with a diagnosis likely indicating chronic pain and at least 2 numeric pain rating scales ≥4; or long-term opioid use (>90 days) [26,27,28].

Negative binomial regression was then used to adjust the relationship between chronic pain and the outcome for potential confounders, including demographics, medical comorbidity using the Charlson index [29], a dichotomous indicator for any inpatient hospitalization during 2020, and psychiatric comorbidities [30]. Regression models involving the count of psychiatric medication changes were further adjusted for the number of psychiatric medications at baseline and whether an opioid or other pain medication was present at baseline. These variables were not included in models involving the number of concurrent CNS-active medications as they are intrinsically part of the outcome measure. A sensitivity analysis was conducted using the same patient selection criteria and outcome definitions but applied to Veterans with PTSD during the calendar year 2012. The purpose of the sensitivity analysis was to determine whether any relationships observed in the primary analysis were stable over time or had changed along with known changes in VA prescription patterns over this period, such as decreases in opioid and benzodiazepine prescription [31,32].

## 3. Results

### 3.1. Patient Selection

In 2020, a total of 637,428 Veterans received care at VA for PTSD, of whom 50.3% (*n* = 320,932) met the criteria for chronic pain. Veterans with PTSD and chronic pain were more likely to be older, female, and African American, relative to those without chronic pain (Table 1). Veterans with chronic pain also displayed higher rates of all psychiatric comorbidities examined, including depressive disorder, substance use disorder, anxiety disorder, bipolar disorder, and psychotic disorder. Veterans with chronic pain were also more likely to be prescribed more antidepressants, opioid analgesics, anticonvulsants, benzodiazepines, antipsychotics, sedative hypnotics, and antimigraine agents.

### 3.2. Medication Changes

**Figure 1:**
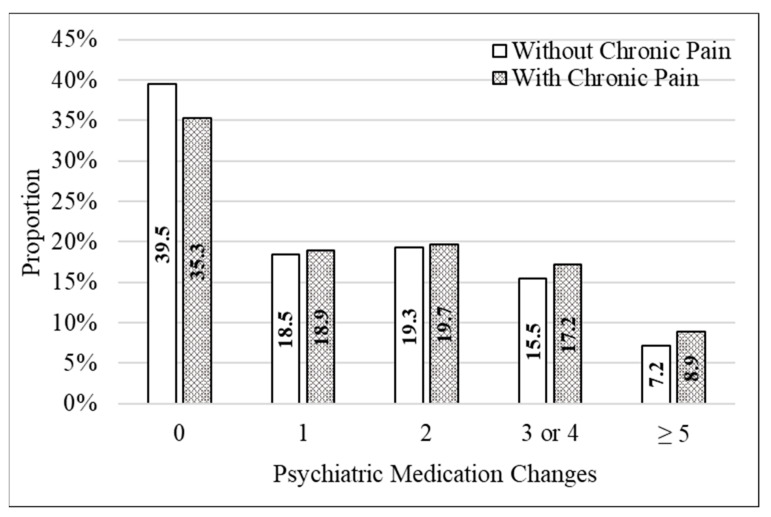
Distribution in the number of changes in CNS medications received by Veterans with PTSD, with and without chronic pain, during 2021.

From 1 January 2021 to 31 December 2021, the number of changes in psychiatric medications was significantly higher for those with chronic pain (mean (M) = 1.8, standard deviation (SD) = 2.0) compared to those without chronic pain (M = 1.6, SD = 1.9) (Z = 38.4, *p* < 0.001). This relationship was also observed with the 2012 sensitivity analysis, where Veterans with chronic pain (M = 2.3, SD = 2.4) had a higher mean number of changes than those without chronic pain (M = 1.9, SD = 2.2) (Z = 58.0, *p* < 0.001). Categorically, 8.9% of Veterans with chronic pain had five or more changes in their psychiatric medication prescriptions compared to 7.2% without chronic pain, and 26.1% of Veterans with chronic pain had three or more changes, compared to 22.7% without chronic pain (Figure 1).

Negative binomial regression was then used to determine whether the relationship between chronic pain and the number of psychiatric medication changes persisted after adjustment for potential confounding factors. The unadjusted IRR was 1.12 (95% CI: 1.11, 1.12), indicating that chronic pain was associated with a 12% greater risk for one additional psychiatric medication change, that is, 12% more likely to have one change than zero changes, 12% more likely to have two changes, relative to one change, etc. After adjustment for important confounding factors including demographics and psychiatric comorbidity, the association between chronic pain and the number of psychiatric medication changes remained significant, and the IRR point estimate was unchanged from the unadjusted model (aIRR = 1.11; 95% CI: 1.10, 1.11; Table 2).

Although not the primary focus of the analysis, several model covariates of note were found to be significantly associated with an elevated risk for changes in psychiatric medications, including female sex, Black or African American race, Charlson Comorbidity Index, recent inpatient hospitalization, and the presence of any examined psychiatric comorbidities. Conversely, covariates associated with a decreased risk for psychiatric medication changes included older age, rural residence, and the presence of at least one psychiatric medication at baseline. The association between chronic pain and an increased risk for the number of psychiatric medication changes was also observed in the 2012 sensitivity analysis (aIIR = 1.16; 95% CI: 1.15, 1.17; Supplemental Table S2).

### 3.3. CNS Polytherapy

**Figure 2:**
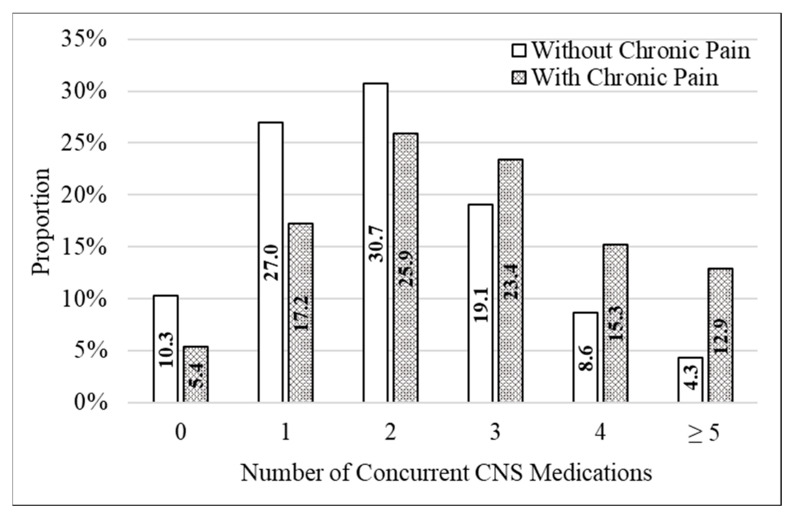
Distribution in the number of concurrent central nervous system (CNS) active medications received by Veterans with PTSD, with and without chronic pain, during 2021.

The number of concurrent CNS-active medications received during the observation period of 1 January 2021 to 31 December 2021 was M = 2.7 (SD = 1.6) for Veterans with chronic pain compared to M = 2.0 (SD = 1.3) for those without chronic pain (Z = 179.7, *p* < 0.001). Categorically, 12.9% of Veterans with chronic pain and PTSD had five or more concurrent CNS medications versus 4.3% without chronic pain (Figure 2). Differences were also found at four concurrent medications (15.3% with chronic pain versus 8.6% without chronic pain) and at three concurrent medications (23.4% with chronic pain and 19.4% without chronic pain). Cumulatively, 51.6% of Veterans with chronic pain were concurrently prescribed three or more CNS-active medications compared to 32.0% of Veterans without chronic pain.

Negative binomial regression was then used to determine whether the relationship between chronic pain and the number of concurrent CNS medications persisted after adjusting for potential confounding factors. The adjusted IRR was 1.29 (95% CI: 1.28, 1.29; Supplemental Table S3), indicating that chronic pain was associated with a 29% greater risk for having one more CNS-active medication. However, when the number of concurrent CNS medications was restricted to just psychiatric medications, the relationship with chronic pain was substantially diminished (aIRR = 1.03; 95% CI: 1.02, 1.03; Supplemental Table S4).

Similar relationships were also observed with the 2012 sensitivity analysis. Veterans with chronic pain (M = 3.1, SD = 1.7) received more concurrent CNS-active medications than Veterans without chronic pain (M = 2.0, SD = 1.4) (Z = 219.1, *p* < 0.001). The association between chronic pain and the number of concurrent CNS medications was observed in the 2012 sensitivity analysis (aIIR = 1.46; 95% CI: 1.45, 1.46; Supplemental Table S5). As seen in the primary analysis, when the number of concurrent CNS medications was restricted to just psychiatric medications, the relationship with chronic pain was substantially diminished (aIRR = 1.07; 95% CI: 1.07, 1.08; Supplemental Table S6).

## 4. Discussion

Our findings, which demonstrate a greater number of psychiatric prescription changes when patients with PTSD also have chronic pain, are consistent with prior work, which demonstrated a magnifying effect of chronic pain on PTSD symptomology [4,5]. We found a moderate effect of a 12% higher risk for each additional medication change across a one-year timeframe among Veterans with PTSD and chronic pain, compared to those with PTSD alone. This higher likelihood of psychiatric medication changes may indicate instability in the patient’s treatment regimen, reflecting a greater symptom burden, instability in symptoms, or greater difficulty in consistently managing symptoms. This finding is also consistent with prior work, which demonstrated an increased number of healthcare visits among Veterans with chronic pain and comorbid PTSD [6]. Veterans with this comorbidity may be seeking, or requiring, a greater number of visits and medication changes in an attempt to treat the heightened symptom load resulting from chronic pain comorbid to PTSD.

Adding to prior work [17], the current findings demonstrate higher rates of CNS polytherapy among Veterans with chronic pain and PTSD, relative to those with only PTSD. The higher rates of CNS-active polytherapy, resulting from the additive effect of psychopharmacologic and analgesic agents, may result, at least in part, from the siloed treatment of these two conditions. Veterans with both conditions may be seen by two different providers, each following a separate set of guidelines [1,33]. Both providers could be prescribing CNS-active medications without knowing the treatment course for the other condition, which could lead to an increased risk for polytherapy in Veterans with chronic pain that is comorbid to PTSD.

As such, providers may benefit from guidelines for treating Veterans with both chronic pain and PTSD. A coordinated cross-specialty treatment plan may result in Veterans having a more stable medication regimen (e.g., fewer medication changes) and lower risks associated with polytherapy [20]. Patients with comorbid chronic pain and PTSD may also benefit from combined behavioral interventions that simultaneously address both PTSD and chronic pain [34]. Because women, rural-dwelling people, and minoritized persons were at greater risk for psychiatric medication changes in our analyses, these populations may stand to benefit most from further research into integrated care for comorbid chronic pain and PTSD.

This study has some limitations. This work includes only Veterans receiving care from VA, so our findings may not generalize to Veterans or other patient populations receiving PTSD care outside of the VA healthcare system. In addition, we were not able to confidently distinguish newly diagnosed PTSD or chronic pain. It is unclear whether the relationships observed in this study would differ between newly diagnosed patients and patients with pre-existing conditions. Another limitation is that certain medications are indicated for both analgesia and psychiatric management (i.e., duloxetine, gabapentin, topiramate). Categorizing these medications as primarily analgesic medications may have impacted the models comparing psychiatric to analgesic prescribing patterns, though this conservative approach was taken to avoid overestimating differences. Finally, the process of prescribing medications in the VA system has been impacted by wide-reaching prescribing initiatives across the past decade, which has significantly reduced overall opioid and benzodiazepine prescriptions [35,36,37]. As such, to examine whether the patterns we identified reflected ongoing clinical phenomenon, as opposed to being in response to healthcare-system-specific initiatives, we conducted sensitivity analyses across time to determine whether the current findings (2021) remained significant a decade prior (2012). The continued significance of our findings supports the consistency of these findings across time. Our focus was to explore, as a proof of concept, the potential impact of chronic pain on the prescription of psychiatric and other CNS medications. Unfortunately, there is no defined value for what constitutes clinically meaningful in the prescribing metrics examined in this study.

In conclusion, the deleterious impact that chronic pain can have on PTSD symptomatology [4,5,6,7] is reflected in the differential prescribing patterns for Veterans with comorbid chronic pain compared to Veterans with PTSD alone. We found that Veterans with comorbid chronic pain and PTSD are at an increased risk for a higher number of medication changes and for receiving CNS-active polytherapy. Providers who treat Veterans with comorbid PTSD and chronic pain may benefit from guidelines to co-manage these conditions, avenues to coordinate care with cross-specialty colleagues, and the development of integrated behavioral interventions that address both conditions.

## Figures and Tables

**Table 1:** Patient characteristics of Veterans with PTSD.

Characteristic	With Chronic Pain N = 320,932 *n* (%)	Without Chronic Pain N = 316,496 *n* (%)
Age		
<40	62,852 (19.6)	89,546 (28.3)
40–54	91,443 (28.5)	83,688 (26.4)
55–64	58,891 (18.4)	39,759 (12.6)
65+	107,746 (33.6)	103,503 (32.7)
Sex		
Male	265,065 (82.6)	272,656 (86.2)
Female	55,867 (17.4)	43,840 (13.9)
Race		
White	188,072 (58.6)	196,678 (62.1)
Black or African American	81,746 (25.5)	68,310 (21.6)
Other or unknown	51,114 (15.9)	51,508 (16.3)
Patient residence		
Urban	263,315 (82.1)	258,079 (81.5)
Rural	57,617 (17.9)	58,417 (18.5)
Comorbidities		
Depressive disorder	208,662 (65.0)	179,329 (56.7)
Substance use disorder	78,613 (24.5)	75,380 (23.8)
Anxiety disorder	46,731 (14.6)	40,999 (13.0)
Bipolar disorder	25,609 (8.0)	23,012 (7.3)
Psychotic disorder	12,640 (3.9)	11,664 (3.4)
CNS medication type		
Antidepressants	202,008 (62.9)	183,241 (57.9)
Anticonvulsants	78,788 (24.6)	43,713 (13.8)
Antipsychotics	38,937 (12.1)	33,547 (10.6)
Opioid analgesics	32,856 (10.2)	2552 (0.8)
Sedative hypnotics	33,852 (10.6)	26,095 (8.2)
Benzodiazepines	18,725 (5.8)	15,404 (4.9)
Anti-migraine agents	11,776 (3.7)	2843 (0.9)
Stimulants	8883 (2.8)	10,007 (3.2)
Anti-Parkinson’s agents	8114 (2.5)	3647 (1.2)
Lithium salts	2737 (0.9)	2628 (0.8)
Non-opioid analgesics	1257 (0.4)	289 (0.1)
Anti-vertigo agents	1179 (0.4)	472 (0.2)
Other CNS-active medications	14,569 (0.9)	5334 (1.7)
Any inpatient hospitalization during 2020		
Yes	44,517 (13.9)	18,806 (5.9)
No	276,415 (86.1)	297,690 (94.1)

CNS = central nervous system; PTSD = post-traumatic stress disorder.

**Table 2:** Clinical characteristics associated with the number of changes in psychiatric medications as a discrete count.

Characteristic	Multivariable Negative Binomial Regression aIRR (95% CI)
Chronic pain	
Not diagnosed	Reference
Diagnosed	1.11 (1.10, 1.11)
Age	
<40	Reference
40–54	0.94 (0.93, 0.94)
55–64	0.84 (0.83, 0.84)
65+	0.67 (0.67, 0.68)
Sex	
Male	Reference
Female	1.14 (1.13, 1.15)
Race	
White	Reference
Black or African American	1.07 (1.06, 1.08)
Other	1.03 (1.02, 1.04)
Patient residence	
Urban	Reference
Rural	0.96 (0.95, 0.97)
Charlson Comorbidity Index	
Per Point	0.99 (0.99, 0.99)
Inpatient hospitalization	
No	Reference
Yes	1.19 (1.17, 1.20)
Psychiatric medications	
0	Reference
1	0.77 (0.77, 0.78)
2	0.78 (0.77, 0.78)
≥3	0.83 (0.82, 0.84)
Comorbidities	
Depressive disorder	1.16 (1.16, 1.17)
Substance use disorder	1.11 (1.10, 1.12)
Anxiety disorder	1.17 (1.15, 1.17)
Psychotic disorder	1.27 (1.25, 1.29)
Bipolar disorder	1.33 (1.31, 1.34)
Pain medication present at baseline	
No pain medication	Reference
Opioid medication	0.97 (0.95, 0.98)
Non-opioid pain medication	0.98 (0.97, 0.99)

aIRR = adjusted incidence rate ratio; CI = confidence interval.

## Data Availability

Not applicable.

## Supplementary Materials

The following supporting information can be downloaded at: https://www.mdpi.com/article/10.3390/jcm12144763/s1, Table S1: CNS active medications typically used for psychiatric indications and observed among dispensed medications. Table S2: Clinical characteristics associated with the number of changes in psychiatric medications as a discrete count for year 2012. Table S3: Clinical characteristics associated with CNS polytherapy medications as a discrete count for year 2021. Table S4: Clinical characteristics associated with psychiatric medications in the CNS polytherapy regimen, as a discrete count for year 2021.Table S5: Clinical characteristics associated with CNS polytherapy medications as a discrete count for year 2012. Table S6: Clinical characteristics associated with psychiatric medications in the CNS polytherapy regimen, as a discrete count for year 2012.
