# Supplementary material for: The Impact of Comorbid Chronic Pain on Pharmacotherapy for Veterans with Post-Traumatic Stress Disorder
Source: J Clin Med. 2023 Jul 19;12(14):4763. doi: 10.3390/jcm12144763 (PMC10380889; doi:10.3390/jcm12144763)
Supplement: Supplementary file 1 [file jcm-12-04763-s001.zip › jcm-2380839-supplementary.pdf]

Supplemental Table S1: CNS active medications typically used for psychiatric indications and observed among dispensed medications.

Class	Medications included
Antidepressants[1]	amitriptyline, amoxapine, clomipramine, desipramine, doxepin, impramine, nortriptyline, protriptyline, trimipramine, phenelzine, selegiline, tranylcypromine, citalopram, escitalopram fluoxetine, fluvoxamine, paroxetine, sertraline, vortioxetine, bupropion, desvenlafaxine, esketamine, levomilnacipran, maprotiline, milnacipran, mirtazapine, nefazodone, trazodone, venlafaxine, vilazodone
Antipsychotics	chlorpromazine, fluphenazine, haloperidol, loxapine, perphenazine, thioridazine, thiothixene, trifluoperazine, aripiprazole, asenapine, brexpiprazole, clozapine, iloperidone, lumateperone, lurasidone, olanzapine, paliperidone, pimavanserin, quetiapine, risperidone, ziprasidone
Anticonvulsants[1]	carbamazepine, divalproex, lamotrigine, valproic acid
Benzodiazepines	alprazolam, chlordiazepoxide, clonazepam, clorazepate, diazepam, estazolam, flurazepam, lorazepam, oxazepam, quazepam, temazepam, triazolam
Z-drug hypnotics	eszopiclone, zaleplon, zolpidem
Stimulants	amphetamine resin complex, dextroamphetamine, lisdexamfetamine, dexmethylphenidate, methylphenidate
Other	buspirone, lithium

[1] Duloxetine, gabapentin, and topiramate were not included among medications typically used for psychiatric medications because they are also commonly used for pain indications.

Supplemental Table S2: Clinical characteristics associated with the number of changes in psychiatric medications as a discrete count for year 2012.

Characteristic	Multivariable negative binomial regression aIRR (95% CI)
Chronic Pain	
Not Diagnosed	Reference
Diagnosed	1.16 (1.15, 1.17)
Age	
< 40	Reference
40-54	0.93 (0.92, 0.94)
55-64	0.78 (0.77, 0.79)
65+	0.71 (0.70, 0.72)
Sex	
Male	Reference
Female	1.11 (1.10, 1.12)
Race	
White	Reference
Black or African American	1.09 (1.08, 1.09)
Other	1.04 (1.03, 1.05)
Patient residence	
Urban	Reference
Rural	0.97 (0.96, 0.98)
Charlson comorbidity index	
Per Point	0.99 (0.98, 0.99)
Inpatient hospitalization	
No	Reference
Yes	1.19 (1.18, 1.20)
Psychiatric medications	
0	Reference
1	0.85 (0.84, 0.86)
2	0.81 (0.81, 0.82)
≥ 3	0.82 (0.81, 0.83)
Comorbidities	
Depressive disorder	1.18 (1.17, 1.18)
Substance use disorder	1.09 (1.09, 1.10)
Anxiety disorder	1.17 (1.15, 1.18)
Psychotic disorder	1.17 (1.16, 1.18)
Bipolar disorder	1.29 (1.28, 1.31)
Pain medication present at baseline	
No pain medication	Reference
Opioid medication	1.02 (1.01, 1.03)
Non-opioid pain medication	1.00 (0.99, 1.01)

aIRR = adjusted incidence rate ratio; CI = confidence interval.

Supplemental Table S3: Clinical characteristics associated with CNS polytherapy medications as a discrete count for year 2021.

Characteristic	Multivariable negative binomial regression aIRR (95% CI)
Chronic Pain	
Not Diagnosed	Reference
Diagnosed	1.29 (1.28, 1.29)
Age	
< 40	Reference
40-54	1.18 (1.18, 1.19)
55-64	1.18 (1.17, 1.19)
65+	1.09 (1.09, 1.10)
Sex	
Male	Reference
Female	1.08 (1.07, 1.08)
Race	
White	Reference
Black or African American	0.87 (0.87, 0.87)
Other	0.95 (0.94, 0.95)
Patient residence	
Urban	Reference
Rural	1.01 (1.01, 1.02)
Charlson comorbidity index	
Per Point	1.02 (1.02, 1.02)
Inpatient hospitalization	
No	Reference
Yes	0.98 (0.97, 0.98)
Comorbidities	
Depressive disorder	1.12 (1.11, 1.12)
Substance use disorder	1.00 (1.00, 1.00)
Anxiety disorder	1.08 (1.08, 1.09)
Psychotic disorder	1.18 (1.17, 1.19)
Bipolar disorder	1.24 (1.24, 1.25)

aIRR = adjusted incidence rate ratio; CI = confidence interval

Supplemental Table S4: Clinical characteristics associated with psychiatric medications in the CNS polytherapy regimen, as a discrete count for year 2021.

**Characteristic**	**Multivariable negative binomial regression aIRR (95% CI)**
Chronic Pain	
Not Diagnosed	Reference
Diagnosed	1.03 (1.02, 1.03)
Age	
< 40	Reference
40-54	1.14 (1.14, 1.14)
55-64	1.15 (1.14, 1.15)
65+	1.09 (1.08, 1.10)
Sex	
Male	Reference
Female	1.03 (1.03, 1.04)
Race	
White	Reference
Black or African American	0.90 (0.90, 0.91)
Other	0.96 (0.95, 0.96)
Patient residence	
Urban	Reference
Rural	0.99 (0.99, 1.00)
Charlson comorbidity index	
Per Point	1.00 (1.00, 1.00)
Inpatient hospitalization	
No	Reference
Yes	0.96 (0.96, 0.97)
Comorbidities	
Depressive disorder	1.12 (1.11, 1.12)
Substance use disorder	0.99 (0.99, 0.99)
Anxiety disorder	1.11 (1.10, 1.11)
Psychotic disorder	1.27 (1.26, 1.28)
Bipolar disorder	1.32 (1.31, 1.33)
Pain medication present at baseline	
No pain medication	Reference
Opioid medication	1.08 (1.07, 1.09)
Non-opioid pain medication	1.14 (1.14, 1.15)

aIRR = adjusted incidence rate ratio; CI = confidence interval

Supplemental Table S5: Clinical characteristics associated with CNS polytherapy medications as a discrete count for year 2012.

Characteristic	Multivariable negative binomial regression aIRR (95% CI)
Chronic Pain	
Not Diagnosed	Reference
Diagnosed	1.46 (1.45, 1.46)
Age	
< 40	Reference
40-54	1.23 (1.22, 1.24)
55-64	1.21 (1.21, 1.22)
65+	1.13 (1.13, 1.14)
Sex	
Male	Reference
Female	1.09 (1.08, 1.09)
Race	
White	Reference
Black or African American	0.87 (0.87, 0.88)
Other	0.95 (0.94, 0.95)
Patient residence	
Urban	Reference
Rural	1.02 (1.02, 1.03)
Charlson comorbidity index	
Per Point	1.02 (1.02, 1.02)
Inpatient hospitalization	
No	Reference
Yes	1.04 (1.04, 1.05)
Comorbidities	
Depressive disorder	1.11 (1.11, 1.12)
Substance use disorder	0.98 (0.98, 0.98)
Anxiety disorder	1.11 (1.10, 1.12)
Psychotic disorder	1.17 (1.17, 1.18)
Bipolar disorder	1.23 (1.22, 1.24)

aIRR = adjusted incidence rate ratio; CI = confidence interval

Supplemental Table S6: Clinical characteristics associated with psychiatric medications in the CNS polytherapy regimen, as a discrete count for year 2012.

**Characteristic**	**Multivariable negative binomial regression aIRR (95% CI)**
Chronic Pain	
Not Diagnosed	Reference
Diagnosed	1.07 (1.07, 1.08)
Age	
< 40	Reference
40-54	1.21 (1.20, 1.21)
55-64	1.22 (1.21, 1.23)
65+	1.17 (1.16, 1.18)
Sex	
Male	Reference
Female	1.05 (1.04, 1.06)
Race	
White	Reference
Black or African American	0.91 (0.91, 0.92)
Other	0.97 (0.96, 0.97)
Patient residence	
Urban	Reference
Rural	1.01 (1.00, 1.01)
Charlson comorbidity index	
Per Point	1.00 (0.99, 1.00)
Inpatient hospitalization	
No	Reference
Yes	1.03 (1.02, 1.03)
Comorbidities	
Depressive disorder	1.12 (1.11, 1.12)
Substance use disorder	0.99 (0.98, 0.99)
Anxiety disorder	1.15 (1.14, 1.15)
Psychotic disorder	1.20 (1.19, 1.20)
Bipolar disorder	1.30 (1.29, 1.30)
Pain medication present at baseline	
No pain medication	Reference
Opioid medication	1.15 (1.15, 1.16)
Non-opioid pain medication	1.15 (1.15, 1.16)

aIRR = adjusted incidence rate ratio; CI = confidence interval
